# Assessment of mortality due to severe SARS-CoV-2 infection in public and private intensive care units in Brazil: a multicenter retrospective cohort study

**DOI:** 10.31744/einstein_journal/2025AO1060

**Published:** 2025-02-28

**Authors:** Thiago Domingos Corrêa, Thais Dias Midega, Ricardo Kenji Nawa, Ricardo Luiz Cordioli, Adriano José Pereira, Moacyr Silva, Bruno de Arruda Bravim, Niklas Soderberg Campos, Amanda Pascoal Valle Felicio, Angelo Antônio Gomes de Carvalho, Andreia Pardini, Raquel Afonso Caserta Eid, Rodrigo Dias Rodrigues, Marcele Liliane Pesavento, Leonardo Van de Wiel Barros Urbano Andari, Bento Fortunato Cardoso dos Santos, Claudia Regina Laselva, Felipe Maia de Toledo Piza, Miguel Cendoroglo, Guilherme de Paula Pinto Schettino, Sidney Klajner, Leonardo José Rolim Ferraz

**Affiliations:** 1 Department of Critical Care Medicine Hospital Israelita Albert Einstein São Paulo SP Brazil Department of Critical Care Medicine, Hospital Israelita Albert Einstein, São Paulo, SP, Brazil.; 2 Department of Critical Care Medicine Hospital Israelita Albert Einstein São Paulo SP Brazil Department of Critical Care Medicine, Hospital Municipal Dr. Moysés Deutsch;Hospital Israelita Albert Einstein, São Paulo, SP, Brazil.; 3 Intensive Care Unit Hospital Israelita Albert Einstein São Paulo SP Brazil Intensive Care Unit, Hospital Municipal da Vila Santa Catarina Dr. Gilson de Cássia Marques de Carvalho;Hospital Israelita Albert Einstein, São Paulo, SP, Brazil.; 4 Department of Critical Care Medicine Hospital Israelita Albert Einstein Goiânia GO Brazil Department of Critical Care Medicine, Hospital Israelita Albert Einstein, Goiânia, GO, Brazil.; 5 Department of Nephrology Hospital Israelita Albert Einstein São Paulo SP Brazil Department of Nephrology, Hospital Israelita Albert Einstein, São Paulo, SP, Brazil.; 6 Instituto Israelita de Responsabilidade Social Hospital Israelita Albert Einstein São Paulo SP Brazil Instituto Israelita de Responsabilidade Social, Hospital Israelita Albert Einstein, São Paulo, SP, Brazil.

**Keywords:** Coronavirus, COVID-19, SARS-CoV-2, Critical care outcomes, Mortality, Intensive care units, Critical care, Coronavirus, infections

## Abstract

**Objective:**

To compare the clinical characteristics, use of organ support, and outcomes of critically ill patients with COVID-19 admitted to public and private intensive care units.

**Methods:**

This multicenter retrospective cohort study included patients admitted to four intensive care units from March 1, 2020, to December 31, 2021. Patients with COVID-19 admitted to public and private intensive care units were compared. The primary outcome of interest, in-hospital mortality, was assessed using a hierarchical logistic regression (multilevel) model adjusted for study site and patient characteristics.

**Results:**

A total of 5,790 patients with COVID-19 were admitted to the participating intensive care units, with 3,321 (57.3%) admitted to private hospitals and 2,469 (42.6%) admitted to public hospitals. Patients in public intensive care units were less likely to be male and had higher median SAPS III scores, Charlson Comorbidity Index values, and SOFA scores. They also required mechanical ventilation (53.1% versus 40.0%, p<0.001), vasopressors (43.1% versus 33.9%, p<0.001), and renal replacement therapy (20.3% versus. 14.5%, p<0.001) more frequently than those in private intensive care units. In contrast, patients in private intensive care units were more frequently managed with non-invasive ventilation (38.0% versus 66.8%; p<0.001) and high-flow nasal cannulas (18.3% versus 48.1%; p<0.001). The in-hospital mortality rate was significantly higher in public intensive care units (40.3%) compared to private intensive care units (16.4%) (adjusted OR=2.96; 95%CI=1.94-4.51; p<0.001).

**Conclusion:**

We observed significant differences in resource utilization and mortality rates between patients with COVID-19 admitted to public and private intensive care units. Patients with COVID-19 in public care units face a higher risk of in-hospital mortality compared to those in private care units.

## In Brief

This retrospective multicenter cohort study compared characteristics and outcomes of 5,790 critically ill patients with COVID-19 in Brazil’s public and private intensive care units. Patients in public intensive care units exhibited greater disease severity, more frequent use of organ support, and higher mortality rates compared to those in private intensive care units. The risk of in-hospital death was more than twice as high in public intensive care units.

## Highlights

■ Public intensive care unit COVID-19 patients presented with more comorbidities and higher severity at admission.

■ Public intensive care units required more invasive organ support (e.g., mechanical ventilation, vasopressors, and renal replacement therapy) but less non-invasive ventilation and high-flow nasal cannula than private intensive care units.

■ In-hospital mortality was higher in public intensive care units, with an increased risk of death even after adjusting for patient characteristics and illness severity at intensive care unit admission

## Graphical abstract

**Figure:**
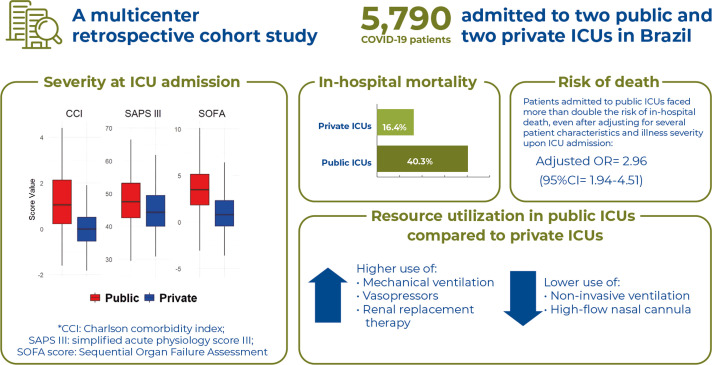

## INTRODUCTION

According to data reported by the World Health Organization (WHO) as of July 2024, the COVID-19 pandemic has infected over 775 million people globally and claimed approximately seven million lives.^1^ The exceptionally high number of severely ill patients requiring organ support and admission to intensive care units (ICUs) has overwhelmed healthcare systems worldwide.^2,3^ The COVID-19 pandemic has exposed significant limitations in critical care disaster management at large centers globally, highlighting the fragility of many countries in effectively responding to such crises, particularly in low- to middle-income countries.^4,5^

Critically ill patients with COVID-19 patients admitted to the ICU present considerable morbidity and mortality, requiring varying degrees of organ support and prolonged ICU and hospital stays.^6^ Nevertheless, despite advances in care throughout the pandemic, improvements in outcomes for severely ill patients have been modest.^7^ This large retrospective study analyzed the characteristics of patients diagnosed with COVID-19 and admitted to Brazilian hospitals, including more than 250,000 cases.^5^The primary finding reported was a high in-hospital mortality rate, even among patients younger than 60 years of age, which was exacerbated by existing regional disparities within the healthcare system.^5^

In Brazil, where over 70% of the population relies on the public healthcare system,^8^ a previous study revealed significant variations in COVID-19 mortality rates between private and public hospitals.^6^Private hospitals had lower proportions of high-risk patients and consequently lower mortality rates, whereas public hospitals had higher proportions of high-risk patients and higher mortality rates.^6^ However, even after adjusting for disease severity, the increased mortality observed in public hospitals persisted, indicating that factors beyond patient-related issues influenced COVID-19 mortality.^6^

Since the design and operation of ICUs across Brazil are heterogeneous,^7^significant challenges arise when comparing the epidemiological characteristics and effectiveness outcomes among patients with COVID-19 in different settings.

## OBJECTIVE

To conduct a comprehensive analysis and comparison of the clinical characteristics, utilization of organ support, and outcomes of critically ill COVID-19 patients admitted to public and private intensive care units in four hospitals in Brazil.

## METHODS

### Study design and oversight

We conducted a multicenter retrospective cohort study involving critically ill adult COVID-19 patients admitted to four ICUs between March 1, 2020, and December 31, 2021. The study was approved by the Institutional Review Board of *Hospital Israelita Albert Einstein* with a waiver for informed consent (CAAE: 65113122.9.0000.0071; # 5.787.083). This study was conducted in accordance with the Strengthening the Reporting of Observational Studies in Epidemiology (STROBE) Statement.^9^

### Setting

This study included critically ill patients admitted to four hospitals in Brazil: two private and two public institutions, all managed by *Hospital Israelita Albert Einstein*. The first hospital (Hospital 1), located in São Paulo, is a private philanthropic quaternary care facility with 724 beds, including 44 medical-surgical adult ICU beds and 95 adult step-down units (SDUs). During the pandemic, the maximum ICU operational capacity for patients with severe COVID-19 increased to 159 beds. The second hospital (Hospital 2), located in Goiânia, is a private philanthropic tertiary care hospital with 35 beds, of which 10 were designated as open medical-surgical adult ICU beds. During the pandemic, the maximum operational capacity of the ICU for patients with severe COVID-19 increased to 40 ICU beds.

The third hospital (Hospital 3), located in São Paulo, is a public secondary care facility with 336 beds, including 60 designated for open medical-surgical adult ICU care. During the pandemic, the total ICU operational capacity for COVID-19 patients increased to 220 beds. The fourth hospital (Hospital 4), also located in São Paulo, is a public secondary care facility with 229 beds, including 30 designated for open medical-surgical adult ICU care. The total ICU operational capacity for patients with severe COVID-19 has increased to 68 beds. Both public hospitals are part of the Public Health System (SUS - *Sistema Único de Saúde*), the Brazilian national healthcare system that provides universal health coverage throughout the country.

### Study participants

Consecutive adult (≥18 years) patients admitted to the participating ICUs between March 1, 2020, and December 31, 2021, with laboratory confirmation of SARS-CoV-2 infection based on a positive reverse-transcriptase-polymerase chain reaction (RT-PCR) assay^(10^ were included. Patients with incomplete data on the following variables were excluded from the analysis: Simplified Acute Physiology Score (SAPS III),^11^ ICU and hospital length of stay (LOS), resource use during ICU stay, and vital status at hospital discharge.

### Patient management

The criteria for ICU admission and the institutional protocol for managing severe SARS-CoV-2 infections have been published elsewhere and are consistent across all four hospitals included in the study.^12,13^

### Data collection and study variables

All study data were retrieved from the Epimed Monitor System^®^ (Epimed Solutions, Rio de Janeiro, Brazil), an electronic structured case report form in which trained ICU case managers prospectively entered patient data.^(14^ Intensive care unit case managers are healthcare professionals with specialized training in critical care data management. To ensure data quality, senior team members conducted regular audits of the data. Collected variables included demographics, comorbidities, admission diagnosis, SAPS 3 score at ICU admission,^11^ Sequential Organ Failure Assessment (SOFA) score at ICU admission,^15^ Charlson Comorbidity Index (CCI),^16^ Modified Frailty Index (MFI),^17^resource use and organ support [vasopressors, non-invasive ventilation (NIV), high-flow nasal cannula (HFNC), mechanical ventilation (MV), renal replacement therapy (RRT) and extracorporeal membrane oxygenation (ECMO)] during ICU stay, destination at hospital discharge, ICU and hospital LOS, and ICU and in-hospital mortality.

### Outcomes

The primary outcome of interest was in-hospital mortality. Secondary outcomes included ICU mortality, ICU LOS, hospital LOS, and the use of organ support during the ICU stay.

### Statistical analysis

Categorical variables were presented as absolute and relative frequencies. Continuous variables were presented as medians with interquartile ranges (IQR). Normality was assessed using the Kolmogorov-Smirnov test.

Comparisons were made between patients with COVID-19 admitted to ICUs in public and private hospitals. Categorical variables were compared using the chi-square test or Fisher’s exact test, as appropriate. Continuous variables were compared using an independent *t*-test or the Mann-Whitney U test for non-normally distributed data.

The primary binary outcome was assessed using a hierarchical logistic regression (multilevel) model adjusted for the study site (random effects at the second level) and for patient characteristics (fixed effects at the first level). The patient-level characteristics included sex, SAPS III score, SOFA score, CCI score, MFI score, and chronic health status. We performed a multicollinearity analysis of the effects included in the model. The results were expressed as adjusted odds ratios (aOR) with 95% confidence intervals (95% CI). We tested the linearity assumption for the continuous variables included in the logistic regression models by analyzing the interaction between each predictor and its logarithm (natural log transformation). When the linearity assumption was violated, continuous variables were categorized.

We performed subgroup analyses for the primary outcome by stratifying patients based on the use of vasopressors, MV, and RRT. To assess whether the effect of ICU type (public versus private) on in-hospital mortality differed across the predefined subgroups, we calculated the p-values for interaction.

Two-tailed tests were used, and statistical significance was defined as p<0.05. Secondary outcome analyses were not adjusted for multiple comparisons. All analyses were conducted using R software version 4.1.0 (R Foundation for Statistical Computing), and GraphPad Prism version 9.5.1 (GraphPad Software Inc., San Diego, CA, USA) was used to plot the graphs.

## RESULTS

### Cohort included

**Figure 1:**
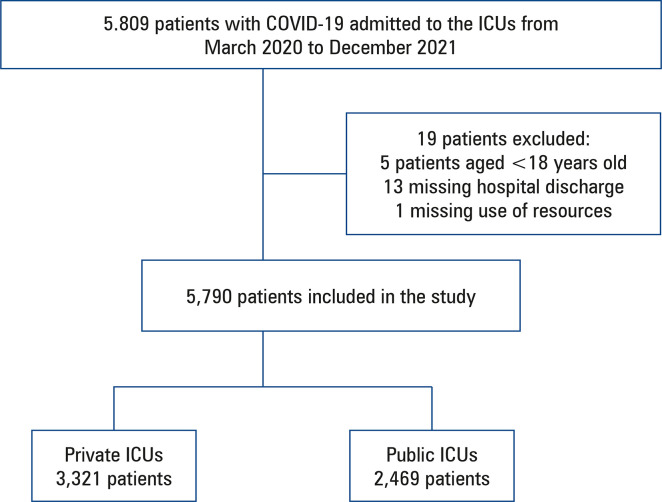
Patients included in the study

ICU: intensive care unit; COVID-19: coronavirus disease 2019.

From March 1, 2020, to December 31, 2021, a total of 5,790 patients with COVID-19 were admitted to the participating ICUs and were included in this analysis. Of these, 3,321 (57.3%) patients were admitted to private hospitals, and 2,469 (42.6%) patients were admitted to public hospitals (Figure 1). Baseline patient characteristics are summarized in table 1.

**Table 1:** Characteristics of patients with COVID-19 admitted to public and private hospitals

Characteristics	All patients (n=5,790)	Public ICUs (n=2,469)	Private ICUs (n=3,321)	p value*
Age, years	61 [49-72]	61 [50-71]	61 [48-74]	0.036^#^
Men, n/total (%)	3,668/5,790 (63.4)	1,379/2,469 (55.9)	2,289/3,321 (68.9)	<0.001^&^
Hospital, n (%)				
1	2,630/5,790 (45.4)	0	2630/3,321 (79.2)	
2	691/5,790 (11.9)	0	691 (20.8)	
3	1,995/5,790 (34.5)	1195/2,469 (48.4)	0	
4	474/5,790 (8.2)	474/2,469 (19.2)	0	
Admission source, n (%)				<0.001^&^
Emergency	3,148/5,790 (54.4)	1,724/2,469 (69.8)	1,424/3,321 (42.9)	
Ward	1,501/5,790 (25.9)	441/2,469 (17.9)	1,060/3,321 (31.9)	
Other ICU	104/5,790 (1.8)	65/2,469 (2.6)	39/3,321 (1.2)	
Intermediate care units	216/5,790 (3.7)	30/2,469 (1.2)	186/3,321 (5.6)	
Others^€^	821/5,790 (14.2)	209/2,469 (8.5)	612/3,321 (18.4)	
SAPS III^$^	46 [42-53]	47 [42-54]	46 [42-52]	<0.001^#^
CCI^%^	0 [0-1]	1 [0-2]	0 [0-1]	<0.001^#^
MFI points^¥^	1 [0-2]	1 [0-2]	1 [0-2]	<0.001^#^
SOFA score	2 [0-4]	3 [1-5]	1 [0-4]	<0.001^#^
Chronic Health Status, n/total (%)				<0.001^&^
Independent	5,103/5,790 (88.1)	2233/2,469 (90.4)	2870/3,321 (86.4)	
Need for assistance	583/5,790 (10.1)	185/2,469 (7.5)	398/3,321 (12.0)	
Restricted to bed	104/5,790 (1.8)	51/2,469 (2.1)	53/3,321 (1.6)	
LOS before ICU admission	1 [0-2]	1 [0-2]	1 [0-2]	0.901^#^
Comorbidities, n/total (%)				
Systemic arterial Hypertension	3,094/4,693 (65.9)	1505/2,055 (73.2)	1589/2,638 (60.2)	<0.001^&^
Diabetes	1,864/4,693 (39.7)	957/2,055 (47.3)	907/2,638 (34.3)	<0.001^&^
Severe COPD	450/4,693 (9.6)	236/2,055 (11.5)	214/2,638 (8.1)	<0.001^&^
Chronic heart failure	355/4,693 (7.6)	193/2,055 (9.4)	162/2,638 (6.1)	<0.001^&^
Chronic renal failure (No Dialysis)	325/4,693 (6.9)	173/2,055 (8.4)	152/2,638 (5.8)	<0.001^&^
Immunosuppression	307/4,693 (6.5)	165/2,055 (8.0)	142/2,638 (5.4)	<0.001^&^
Locoregional cancer	338/4,693 (7.2)	132/2,055 (6.4)	206/2,638 (7.8)	0.078^&^
Asthma	278/4,693 (5.9)	108/2,055 (5.3)	170/2,638 (6.4)	0.099^&^
Chronic renal failure (with Dialysis)	144/4,693 (3.1)	73/2,055 (3.6)	71/2,638 (2.7)	0.107^&^
Metastatic cancer	76/4,693 (1.6)	41/2,055 (2.0)	35/2,638 (1.3)	0.092^&^
Hematological cancer	98/4,693 (2.1)	26/2,055 (1.3)	72/2,638 (2.7)	0.001^&^

Data are presented as median and interquartile range [IQR] or n/n total (%). The percentages may not be 100% due to rounding.

* p-values were calculated using ^#^ Mann-Whitney U test and ^&^ χ^2^ test; ^$^ The SAPS III score ranges from 0 to 217, with higher scores indicating more severe illness and a higher risk of death; ^%^ Charlson comorbidity index is based on a point scoring system (from 0 to 37) that predicts 10-year survival in patients with multiple comorbidities. A score of zero indicates no comorbidities, while a higher score predicts a greater likelihood of mortality; ^¥^ Modified frailty index ranges from 1 to 11, with 1 point assigned for each of up to 11 possible frailty components (comorbidities or deficits); ^€ “^Others” includes cases where patients were admitted from the operating room, transferred from another hospital or institution or for unspecified reasons.

CCI: Charlson Comorbidity Index; MFI: modified frailty index; SAPS III: simplified acute physiology score III; COPD: chronic obstructive pulmonary disease; SOFA score: Sequential Organ Failure Assessment; ICU: intensive care units; LOS: length of stay.

Critically ill patients with COVID-19 admitted to public ICUs differed significantly from those in private ICUs. Patients in public ICUs were less frequently male (55.9% *versus* 68.9%; p<0.001), and had higher median [IQR] SAPS III scores (47 [42-54] *versus* 46 [42-52]; p<0.001), CCI (1 [0-2] *versus* 0 [0-1]; p<0.001) and SOFA scores (3 [1-5] *versus* 1 [0-4]; p<0.001) compared to those in private ICUs (Table 1).

Patients in public ICUs also presented with a higher prevalence of comorbid conditions compared to those in private ICUs: systemic arterial hypertension (73.2% *versus* 60.2%; p<0.001), diabetes (47.3% *versus* 34.3%; p<0.001), severe chronic obstructive pulmonary disease (11.5% *versus* 8.1%; p<0.001), chronic heart failure (9.4% *versus* 6.1%; p<0.001), chronic renal failure without dialysis (8.4% *versus* 5.8%; p<0.001), immunosuppression (8.0% *versus* 5.4%; p<0.001) and hematologic cancer (2.7% *versus* 1.3; p<0.001).

Hospital LOS before ICU admission did not differ significantly between public and private hospitals (1 [0-2] *versus* 1 [0-2] days; p=0.901). Most patients in both public and private ICUs were admitted from the emergency department or hospital wards (Table 1).

### Outcomes and resource use

In-hospital mortality was significantly higher in critically ill patients with COVID-19 admitted to public ICUs (40.3%) compared to those in private ICUs (16.4%) (aOR: 2.96; 95%CI=1.94-4.51; p<0.001) (Tables 2 and 3). Similarly, ICU mortality rates were higher for patients in public ICUs (37.0% *versus* 15.6%; p<0.001) (Table 2). While ICU LOS did not differ significantly between public and private hospitals (9 [4-18] *versus* 9 [4-18] days, p=0.503), the overall hospital LOS was shorter for patients in public hospitals (13 [8-24] *versus* 14 [9-25] days, p=0.001) (Table 2).

**Table 2:** Outcomes and resource use of studied patients and comparisons between patients with COVID-19 admitted to private and public intensive care units

Outcomes	All patients (n=5,790)	Public ICUs (n=2,469)	Private ICUs (n=3,321)	p value*
Hospital mortality, n (%)	1,540/5,790 (26.6)	994/2,469 (40.3)	546/3,321 (16.4)	<0.001^&^
ICU mortality, n (%)	1,430/5,790 (24.7)	913 /2,469 (37.0)	517/3,321 (15.6)	<0.001^&^
ICU LOS	9 [4-18]	9 [4-18]	9 [4-18]	0.503^#^
Hospital LOS	14 [8-24]	13 [8-24]	14 [9-25]	0.001^#^
Support during ICU stay, n (%)				
Vasopressors	2,190/5,790 (37.8)	1,063/2,469 (43.1)	1,127/3,321 (33.9)	<0.001^&^
MV	2,640/5,790 (45.6)	1,312/2,469 (53.1)	1,328/3,321 (40.0)	<0.001^&^
NIV	3,158/5,790 (54.5)	938/2,469 (38.0)	2,220/3,321 (66.8)	<0.001^&^
RRT	981/5,790 (16.9)	501/2,469 (20.3)	480/3,321 (14.5)	<0.001^&^
HFNC	2,047/5,790 (35.4)	451/2,469 (18.3)	1,596/3,321 (48.1)	<0.001^&^
ECMO	48/5,790 (0.8)	0/2,469 (0.0)	48/3,321 (1.4)	<0.001^&^
MV duration	11 [6-21]	11 [5-18]	12 [7-25]	<0.001^#^
Tracheotomy	455/5,790 (7.9)	151/2,469 (6.1)	304/3,321 (9.2)	<0.001^&^

Data are presented as median and interquartile range [IQR] or n/n total (%). The percentages may not be 100% due to rounding.

*p values were calculated using ^#^ Mann-Whitney U test and ^&^ χ^2^ test.

ECMO: extracorporeal membrane oxygenation; ICU: intensive care unit; MV: mechanical ventilation; LOS: length of stay; RRT: renal replacement therapy; NIV: non-invasive ventilation; HFNC: high-flow nasal cannulas.

**Table 3:** Results of the hierarchical logistic regression (multilevel) analysis for in-hospital mortality

Fixed effects	OR	(95%CI)	p value
Male sex	1.19	1.03-1.38	0.016
SAPS III score*			
<42	Reference		
43-46	1.53	1.21-1.93	<0.001
47-53	2.07	1.68-2.54	<0.001
54-98	3.48	2.79-4.34	<0.001
SOFA score	1.22	1.19-1.25	<0.001
Charlson Comorbidity index	1.10	1.04-1.15	<0.001
MFI	1.14	1.06-1.23	<0.001
Chronic Health Status			
Independent	Reference		
Need assistance	1.70	1.33-2.17	<0.001
Restricted to bed	1.25	0.78-2.00	0.348
Public ICU	2.96	1.94-4.51	<0.001

* The SAPS III score was categorized according to percentiles because the linearity assumption was violated.

OR: odds ratio; 95%CI: 95% confidence interval; MFI: modified frailty index; SAPS III: simplified acute physiology score III; SOFA score: Sequential Organ Failure Assessment; ICU: intensive care units.

Patients in public ICUs required more intensive resource use compared to those in private ICUs: MV 53.1% *versus* 40.0%, vasopressors 43.1% *versus* 33.9%, RRT 20.3% *versus* 14.5% (all p<0.001).

Conversely, the use of non-invasive ventilation (NIV) and high-flow nasal cannulas (HFNC) was less frequent in public ICUs: NIV 38.0% *versus* 66.8% (p<0.001) and HFNC 18.3% *versus* 48.1% (p<0.001). The median [IQR] duration of MV was shorter among patients in public ICUs compared to private ICUs (11 [5-18] *versus* 12 [7-25] days; p<0.001) (Table 2).

### Subgroup analyses

**Figure 2:**
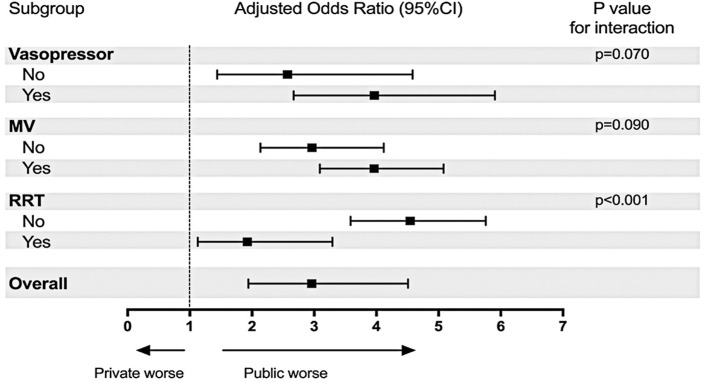
Primary outcome in the pre-specified subgroup analyses

Patients with COVID-19 admitted to a public ICU who received vasopressors and/or MV, and RRT exhibited higher in-hospital and ICU mortality rates, as well as shorter ICU and hospital LOS compared to those receiving the same organ support in a private ICU (Table 4). Heterogeneity in the treatment effect on in-hospital mortality was observed among the subgroup of patients based on the receipt of RRT (p<0.001 for interaction) (Figure 2).

**Table 4:** Outcomes between patients with COVID-19 admitted to public and private hospitals according to the use of mechanical ventilation, vasopressors, and renal replacement therapy

Outcomes	All patients (n=5,790)	Public ICUs (n=2,469)	Private ICUs (n=3,321)	p value*
According to the use of vasopressors				
Received vasopressors	2,190	1,063	1,127	
ICU mortality	1104/2,190 (50.4)	707/1,063 (66.5)	397/1,127 (35.2)	<0.001^&^
Hospital mortality	1146/2,190 (52.3)	736/1,063 (69.2)	410/1,127 (36.4)	<0.001^&^
ICU LOS, days	18.00 [11.00, 29.00]	15 [8-25]	20 [13-33]	<0.001^#^
Hospital LOS, days	23.00 [14.00, 36.00]	19 [11-31]	27 [17-42]	<0.001^#^
Did not receive vasopressors	3,600	1406	2,194	
ICU mortality	326/3,600 (9.1)	206/1,406 (14.7)	120/2,194 (5.5)	<0.001^&^
Hospital mortality	394/3,600 (10.9)	258/1,406 (18.3)	136/2,194 (6.2)	<0.001^&^
ICU LOS, days	6 [3-10]	6 [3-11]	6 [3-10]	0.032^#^
Hospital LOS, days	11 [7-17]	11 [6-18]	11 [7-16]	0.888^#^
According to the use of MV				
Received MV	2,640	1,312	1,328	
ICU mortality	1267/2,640 (48.0)	818/1,312 (62.3)	449/1,328 (33.8)	<0.001^&^
Hospital mortality	1309/2,640 (49.6)	852/1,312 (64.9)	457/1,328 (34.4)	<0.001^&^
ICU LOS, days	17 [10-28]	15 [8-24]	19 [13-31]	<0.001^#^
Hospital LOS, days	22 [13-35]	18 [11-30]	25 [16-40]	<0.001^#^
Did not receive MV	3,150	1157	1993	
ICU mortality	163/3,150 (5.2)	95/1,157 (8.2)	68/1,993 (3.4)	<0.001^&^
Hospital mortality	231/3,150 (7.3)	142/1,157 (12.3)	89/1,993 (4.5)	<0.001^&^
ICU LOS, days	5 [3-9]	5.00 [3-9]	5 [3-8]	0.701^#^
Hospital LOS, days	10 [7-15]	10 [6-16]	10 [7-14]	0.104^#^
According to the use of RRT				
Received RRT	981	501	480	
ICU mortality	641/981 (65.3)	349/501 (69.7)	292/480 (60.8)	<0.001^&^
Hospital mortality	657/981 (67.0)	361/501 (72.1)	296/480 (61.7)	<0.001^&^
ICU LOS, days	21 [12-35]	17 [9-28]	27 [17-42]	<0.001^#^
Hospital LOS, days	25 [14-42]	20 [12-33]	31 [20-53]	<0.001^#^
Did not receive RRT	4,809	1,968	2,841	
ICU mortality	789/4,809 (16.4)	564/1,968 (28.7)	225/2,841 (7.9)	<0.001^&^
Hospital mortality	883/4,809 (18.4)	633/1,968 (32.2)	250/2,841 (8.8)	<0.001^&^
ICU LOS, days	8 [4-14]	8 [4-15]	7 [4-14]	0.133^#^
Hospital LOS, days	12 [8-21]	12 [7-21]	13 [8-20]	0.037^#^

Data are presented as median and interquartile range [IQR] or n/n total (%). The percentages may not be 100% due to rounding.

*p values were calculated using ^#^Mann-Whitney U test and ^&^χ^2^ test.

ICU: intensive care unit; MV: mechanical ventilation; NIV: non-invasive ventilation; LOS: length of stay; RRT: renal replacement therapy.

The x-axis represents the adjusted odds ratio. Values >1.0 indicate an increased risk of in-hospital mortality in patients admitted to public ICUs compared to patients admitted to private ICUs. The 95%CIs were not adjusted for multiple comparisons. 95%CI: 95% confidence interval; MV: mechanical ventilation; RRT: renal replacement therapy.

## DISCUSSION

In this multicenter retrospective cohort study, we reported the clinical characteristics, resource utilization, and outcomes of 5,790 patients admitted to four ICUs in Brazil over a 2-year period. Notably, our study identified significant disparities between public and private ICUs in Brazil. Patients with COVID-19 admitted to public ICUs presented with greater severity at the time of ICU admission, required more intensive organ support, and exhibited a higher risk of in-hospital mortality compared to those admitted to private ICUs.

The overall in-hospital mortality rate in our cohort of patients with COVID-19 was lower than that reported in other Brazilian studies.^5,6^ This reduced mortality rate related to factors specific to the participating ICUs and hospitals, including organizational factors, availability of human resources and organ support devices, staffing patterns, and economic and social disparities across the country. Marked disparities in resource availability and outcomes for hospitalized patients with COVID-19 across different states of Brazil have been previously reported.^(5,6,18,19^

In our study, in-hospital mortality was higher among patients with COVID-19 admitted to public ICUs compared to those admitted to private ICUs. This disparity persisted even after adjusting for several patient characteristics and illness severity upon ICU admission. The observed differences in COVID-19 mortality between public and private ICUs can be attributed to a combination of factors, including patient characteristics not fully captured by severity scores, such as epidemiological factors, performance status, and socioeconomic conditions, as well as organizational aspects related to the participating ICUs and hospitals. Our findings align with those of a previous study conducted in Brazil,^6^ which demonstrated that private hospitals had a lower mortality rate among patients with COVID-19 than public hospitals.

In our study, patients with COVID-19 admitted to public ICUs exhibited a greater number of comorbidities and higher in-hospital mortality rates compared to those admitted to private ICUs. Our findings are consistent with those of a previous meta-analysis, which demonstrated that the presence of comorbidities worsens the prognosis of patients with COVID-19.^20^ Systemic arterial hypertension, diabetes, cardiovascular diseases, and respiratory conditions have all been associated with a poor prognosis.^20^ Furthermore, it is important to note that some patients in our cohort may have had undiagnosed comorbidities and/or poorly controlled conditions that could have adversely impacted their outcomes. This effect may be particularly pronounced in patients from lower socioeconomic backgrounds, who may have limited access to regular healthcare and preventive services.^21^

Moreover, a meta-analysis demonstrated a strong correlation between lower socioeconomic factors, such as income, level of education, employment, housing quality, and urbanicity, and COVID-19 outcomes, which is particularly pronounced among racial and ethnic minority groups.^21^ Increased mortality among patients with COVID-19 has also been observed in the northern region of Brazil and among the Pardo and Black populations.^22^ Although our study did not specifically address the effect of socioeconomic characteristics on clinical outcomes, it is important to consider the context of the Brazilian healthcare system. Patients admitted to public hospitals during the COVID-19 pandemic may have faced more challenging socioeconomic conditions, which could have contributed to their poorer underlying health.

A previous Brazilian study reported that hospitals with more experienced ICU staff had better clinical outcomes during the COVID-19 pandemic.^6^ In our cohort, both public and private ICUs had to markedly expand their operational capacity to accommodate critically ill patients with COVID-19. Nevertheless, public hospitals increased their capacity proportionally more than private hospitals, which may have resulted in less experienced staff teams in the former. This disparity in ICU staff patterns may have contributed to the worse clinical outcomes observed in public ICUs compared to private ICUs in our study.

In our study, we observed that patients admitted to public ICUs received MV, vasopressors, and RRT more frequently, while non-invasive ventilatory support, such as NIV and HFNC, was utilized less frequently compared to patients admitted to private ICUs. These findings likely reflect the higher severity of illness among patients in public ICUs, necessitating more frequent use of invasive support rather than indicating a shortage of resources within the public ICUs included in our study. However, a previous study documented resource limitations in Brazilian public ICUs,^18^ noting that NIV and HFNC were more commonly available in private hospitals than in public hospitals.^18^ The use of NIV and HFNC has been previously associated with lower risks of intubation and MV.^23,24^

We found that the LOS before ICU admission did not differ between private and public hospitals. However, emergency department (ED) admissions were more common in public ICUs, while hospital admissions were more prevalent in private ICUs. We hypothesized that patients in our study may have been admitted earlier in private hospitals compared to public hospitals. Consequently, the time interval between the onset of COVID-19 and the start of treatment may have been longer for patients admitted to public hospitals than for those admitted to private hospitals, potentially impacting clinical outcomes. Indeed, an experimental model of abdominal sepsis has demonstrated that delays in treatment initiation between sepsis onset and the initiation of resuscitation are associated with worse outcomes.^25^ However, our data precluded us from testing this hypothesis.

Future research should address the disparities observed between public and private healthcare settings in the management of COVID-19. Studies focusing on healthcare system access, resource distribution, and utilization across different types of ICUs are crucial for evaluating strategies to improve care. These investigations will inform evidence-based policies aimed at reducing healthcare inequalities and enhancing outcomes for all patients during crises such as the COVID-19 pandemic.

Our study has some limitations. First, due to data collection constraints during the COVID-19 pandemic, we were unable to identify patients receiving palliative care during their ICU stay, which may have influenced our outcomes. Second, while we adjusted our primary analysis for several baseline patient characteristics, such as chronic health status, CCI, MFI, SAPS III, and SOFA scores, these severity scores may not fully capture the severity of COVID-19 in our study.^26^ Third, we did not account for the different phases of the COVID-19 pandemic in Brazil, including various waves and the introduction of treatments and vaccines, which may have influenced patient outcomes.^27-29^ Fourth, although our study included four different hospitals, all of these facilities were managed by the *Hospital Israelita Albert Einstein*, which may limit the generalizability of our results to other healthcare settings. Finally, we lacked data on specific organizational factors and resource allocation in ICUs during the COVID-19 pandemic, such as staffing adjustments and variations in strain between units. Future studies should incorporate these factors to gain a more comprehensive understanding of ICU management during health crises.

## CONCLUSION

In conclusion, our study revealed significant disparities in outcomes of patients with COVID-19, between public and private intensive care units in Brazil. Specifically, patients with COVID-19 admitted to public intensive care units face a higher risk of in-hospital death compared to those treated in private care units. These findings support the implementation of evidence-based policies aimed at improving outcomes for critically ill patients and highlight the need for researchers to address the socioeconomic and organizational factors that contribute to inequalities within the Brazilian healthcare system.
